# Retrospective analysis of decision-making in post-traumatic posterior shoulder instability

**DOI:** 10.1007/s00264-023-06045-9

**Published:** 2023-12-04

**Authors:** Simon Bovenkerk, Carsten Englert

**Affiliations:** 1 Department of Trauma Surgery, University Hospital Regensburg, Franz-Josef-Strauß-Allee 11, 93053 Regensburg, Germany; 2 Department of Orthopedic and Trauma Surgery, Hospital Zum Heiligen Geist Fritzlar, Am Hospital 6, 34560 Fritzlar, Germany

**Keywords:** Posterior shoulder instability, Shoulder dislocation, Arthroscopy, Conservative therapy, Western Ontario Shoulder Instability Index (WOSI), Decision-making

## Abstract

**Purpose:**

This study aims to assess the clinical outcomes in the management of post-traumatic posterior shoulder instability (PSI) with a focus on the decision-making process for operative and conservative treatments.

**Introduction:**

PSI can result from traumatic events, impacting a patient’s quality of life. This study delves to better indicate decision-making for operative indication of post-traumatic PSI patients.

**Methods:**

Patients who sustained posterior shoulder dislocations were selected from a single surgeon’s database within a five-year period. Cases of degenerative or genetically caused PSI were excluded, resulting in a cohort of 28. Patients were initially managed conservatively but indicated for surgery if they were unable to actively stabilize the shoulder or exhibited bony or cartilage defects confirmed through imaging. If conservative treatment did not yield significant improvements, it was classified as a failure, and operative intervention was recommended. The WOSI Score, ROM, and X-ray were employed to evaluate the success of treatment.

**Results:**

Out of the 28 patients, 11 received conservative, seven immediate surgeries, and ten transitioned from conservative to operative treatment. The overall success rate showed 25 good to excellent results. In the persistent conservative treatment group, the initial WOSI score was significantly lower compared to the operative group.

**Conclusion:**

This study suggests that post-traumatic PSI can be successfully managed conservatively with initial low clinical symptoms (low WOSI score) and in the absence of absolute indications for operative treatment. When surgery is necessary, arthroscopic procedures proved effective in achieving good to excellent results in 16 out of 17 cases.

## Introduction

Participation in contact sports or traumatic incidents, particularly those involving powerful rearward forces or leverages on the shoulder joint, can lead to traumatic posterior shoulder dislocation and instability (PSI) [1, 2]. Among these injuries, posterior labrum lesions account for the majority, constituting 50% of all detected posterior shoulder instability cases [3]. Bony lesions, such as humeral head impressions on track fractures or glenoid rim fractures, make up another 25% [3]. In the treatment of such traumatic cases, restoring stability by addressing soft tissue, bone, and cartilage defects, as well as the posterior joint capsule, is of paramount importance [3]. Both nonoperative and operative techniques have demonstrated varying degrees of success.

Success rates for conservative treatment of PSI span a wide range, with reported rates ranging from 16 to 68% [1, 4–6]. Blacknall and Moroder, for instance, have illustrated high success rates with conservative treatments, including physiotherapy and electric muscle stimulation (EMS) [7–9]. Moroder’s studies suggest that conservative treatment yields high success rates, particularly when focusing on muscle strengthening and motor control movement re-education in patients with repetitive microtrauma (29%) or atraumatic origin (71%) [7, 10]. Festbaum goes a step further by asserting that conservative therapy can be a viable option even for patients with an acute traumatic posterior shoulder dislocation [11]. In general, an attempt at conservative therapy should be initiated unless there are essential operative indications, such as bony or chondral defects leading to sub- or dislocation, an inability to actively centre the glenohumeral joint during motion, or chronic dislocation. The persistence of PSI or pain beyond three months of conservative treatment signifies conservative treatment failure, and surgery is considered the next line of action to address underlying morphological pathology [12, 13].

When surgery is warranted, arthroscopic operative methods have demonstrated their superiority over open surgery and have become the gold standard, particularly in cases of trauma or microtrauma in athletes, addressing both soft tissue and bony lesions [2, 3, 14–17]. Arthroscopic treatment of PSI is associated with lower rates of recurrent instability, shorter recovery times, improved cosmetic outcomes, fewer complications, and better results compared to open surgery, particularly for athletes [3, 14, 16]. Certain risk factors have been identified, such as the presence of chondral damage, prior or concurrent shoulder surgeries, and workman’s compensation claims [17, 18].

To date, only one study has specifically compared nonoperative and operative arthroscopic treatments for PSI.

Considering the results reported by Cruz-Ferreira et al. the hypothesis driving this case–control study is that nonoperative therapy, when appropriately indicated, can yield results comparable to those achieved through operative treatment. As such, this study retrospectively analyzes the decision-making process and outcomes of 28 patients with post-traumatic posterior shoulder instability. The assessment is based on the type of treatment received (non-operative vs. operative), patient satisfaction to begin and after therapy measured by the Western Ontario Shoulder Instability Index (WOSI), clinical examination encompassing range of motion and strength, as well as X-ray findings.

## Materials and methods

Institutional Review Board (IRB) approval was obtained for this study under protocol number 20–1930-101. Patients with the International Classification of Diseases (ICD) codes S43.00 (unspecified shoulder dislocation) and S43.02 (shoulder dislocation to the rear) were identified from the records of a single surgeon. This data extraction was facilitated using the Tomedo software, covering the period from 2016 to 2020, resulting in a total of 353 patients with these specific ICD codes.

**Fig. 1:**
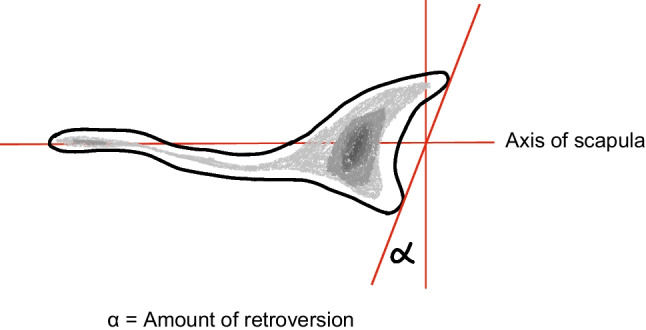
Determination of the angle of glenoid version (α), visualizes the procedure for determining the angle (α) using the method of Friedman et al. [19]. A line is drawn through the body of the shoulder blade as the axis of scapula. On this axis, an orthogonal is drawn as a reference line. A third line forms a tangent to the glenoid surface. The glenoid version and thus the angle (α) are ultimately formed by the intersection of the orthogonal and the tangent

For every patient with a suspected diagnosis of posterior shoulder instability (PSI), an initial imaging examination was conducted, which included native X-rays, MRI, and, when deemed necessary, CT scans. The findings from these imaging studies played a pivotal role in determining the appropriate course of therapy, with due consideration of the entirety of the diagnostic data. As part of this study, the MRI, and CT datasets were meticulously reevaluated to eliminate the possibility of initially missing information and to monitor the development of any osteoarthritic changes in follow-up assessments. Additionally, we investigated whether the glenoid version might have an impact on PSI. To measure the glenoid version, we employed the methods proposed by Friedman et al. and Poon et al. [19, 20], which are based on 2D CT scans of the scapula and humerus (Fig. 1).

The Western Ontario Shoulder Instability Index (WOSI) was used in this study to assess patients. The WOSI comprises four main groups: physical symptoms, sport/recreation/work, lifestyle, and emotions. These groups encompass a total of 21 questions, each rated on a visual analogue scale ranging from zero to ten points. The highest possible score, indicating the most severe symptoms, is 210 points, while the best possible score is zero points.

Two separate WOSI scores were collected: one at the beginning of the treatment period and another between April and October 2020. These scores were structured to facilitate a comparison of the patient’s shoulder condition before and after treatment. A statistical analysis was carried out using Prism 10.0. Among the grouped and matched parameters for the condition operation and the status pre- and post-treatment a normal distribution was proved. Overall difference in the groups was assessed by analysis of variance (ANOVA) followed by post hoc comparisons made by Tukey’s test. Throughout, statistical significance was accepted for *p* < 0.05.

To enable comparability with future studies, we classified our patients based on the ABC scheme developed by Moroder et al. [21]. Moroder’s classification system for posterior glenohumeral instability (PGHI) categorizes patients into three types from A to C, based on the underlying mechanism. Type A includes patients who experienced acute shoulder dislocation followed by instability for the first time. Patients with recurring dynamic posterior instability, occurring in various phases of movement, are assigned to group B. Group C encompasses patients with chronic static PGHI. This classification aids in characterizing the study population for future research comparisons.

## Results

Between 2016 and 2021, a total of 353 patients were treated with the ICD codes S43.00 or S43.02, of which 33 met the criteria for posterior shoulder instability (PSI). Of these 33 patients, 28 were identified as having trauma-induced PSI, and all 28 of them participated in the survey. The gender distribution among these patients was seven females and 21 males. The mean age of the cohort was 33 years, ranging from 17 to 55 years, and the average duration of treatment was 33.11 months, with treatment durations ranging from four to 66 months. Among the patients, 15 experienced shoulder instability on their right side, nine on their left, and four had bilateral shoulder problems. Using the Friedman method, the average glenoid retroversion was found to be − 4.73°, while the “glenoid vault” method yielded an average retroversion of − 17.53°. Of the 28 patients, 17 received operative treatment, all of which were performed arthroscopically. Initially designated for non-operative therapy, 11 out of 21 patients were successfully treated nonoperative.

Primarily, operative treatment was indicated for seven patients, while ten patients transitioned from nonoperative to operative treatment within three months. In the operative treatment group, every patient displayed bony damage on MRI or during arthroscopic procedures. During clinical testing, the posterior apprehension test was positive in ten cases, the Kiebler test in nine cases, and the Habermeyer test in five cases. For ten patients, conservative therapy proved unsuccessful, necessitating a shift to arthroscopic intervention. According to the ABC classification for diagnosis and pathogenesis by Moroder, 14 of the arthroscopically treated patients were classified under group A2 (acute posterior dislocation), primarily stemming from a single traumatic event. One patient, numbered as patient one, was classified under group B2 (structural dynamic posterior instability), characterized by a SLAP and Bankart lesion that enabled the patient to actively dislocate their shoulder posteriorly. Patient number 13 displayed hyperlaxity, with a Beighton score of six, and an additional reverse Bankart lesion from a prior untreated primary dislocation six years earlier, classifying them under group B2 (structural dynamic posterior instability). Patient 17 experienced shoulder dislocation during a backward-facing fall with sudden abduction and extension, placing them in group B1 (Fig. 2).

**Fig. 2.1–2.2:**
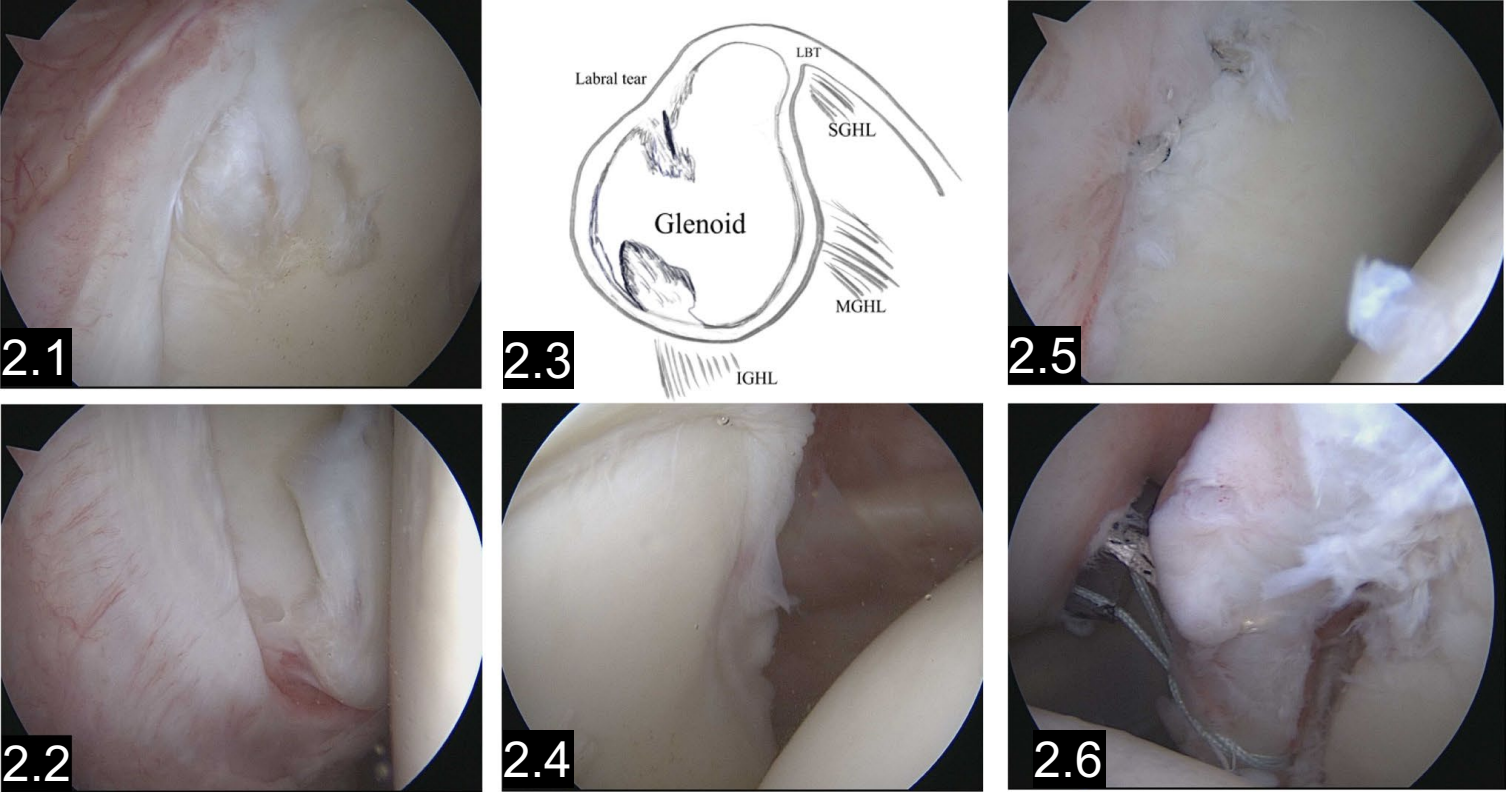
Arthroscopic confirmation of an almost complete posterior labral lesion from 6 to 11:45. The labrum is torn off, and two broader fibers hang over the glenoid rim into the joint space. A schematic scene in Fig. 2.3 illustrates the labral defect. Figure 2.4 A healthy labral complex from 12 to 3 o clock on the glenoid rim is visible. Figure 2.5–2.6 Anatomical reconstruction of the posterior labral lesion with reinforcement threads to the glenoid rim at ten o’clock and placement of sutures for further fixation with anchors are visible at 7 o’clock through the torn labrum. SGHL superior glenohumeral ligament, MGHL medial glenohumeral ligament, IGHL inferior glenohumeral ligament, LBT long biceps tendon

On average, patients treated arthroscopically received a total of 2.0 anchors, corresponding to approximately one anchor for every sixth of the glenoid rim defect.

The pathology within the conservative group was assessed using MRI examinations, revealing a total of six injuries to the bone or soft tissue. Within the conservative cohort, ten patients belonged to group A2 (acute posterior dislocation), and one patient to group A1. Group A1 includes patients with an acute posterior subluxation, as in the case of patient number 19, who suffered repeated microtrauma from Thai boxing. In two cases (patient number 18 and 25), with an A2 classification, only conservative treatment was carried out at the patient’s request. For one patient, who had experienced a reverse multifragmentary Bankart fracture due to a motorcycle accident, the treatment involved a bone block harvested from the iliac crest and transferred to the posterior glenoid rim through an arthroscopic procedure with mini open portal to place the bone block (Fig. 3).

**Fig. 3.1:**
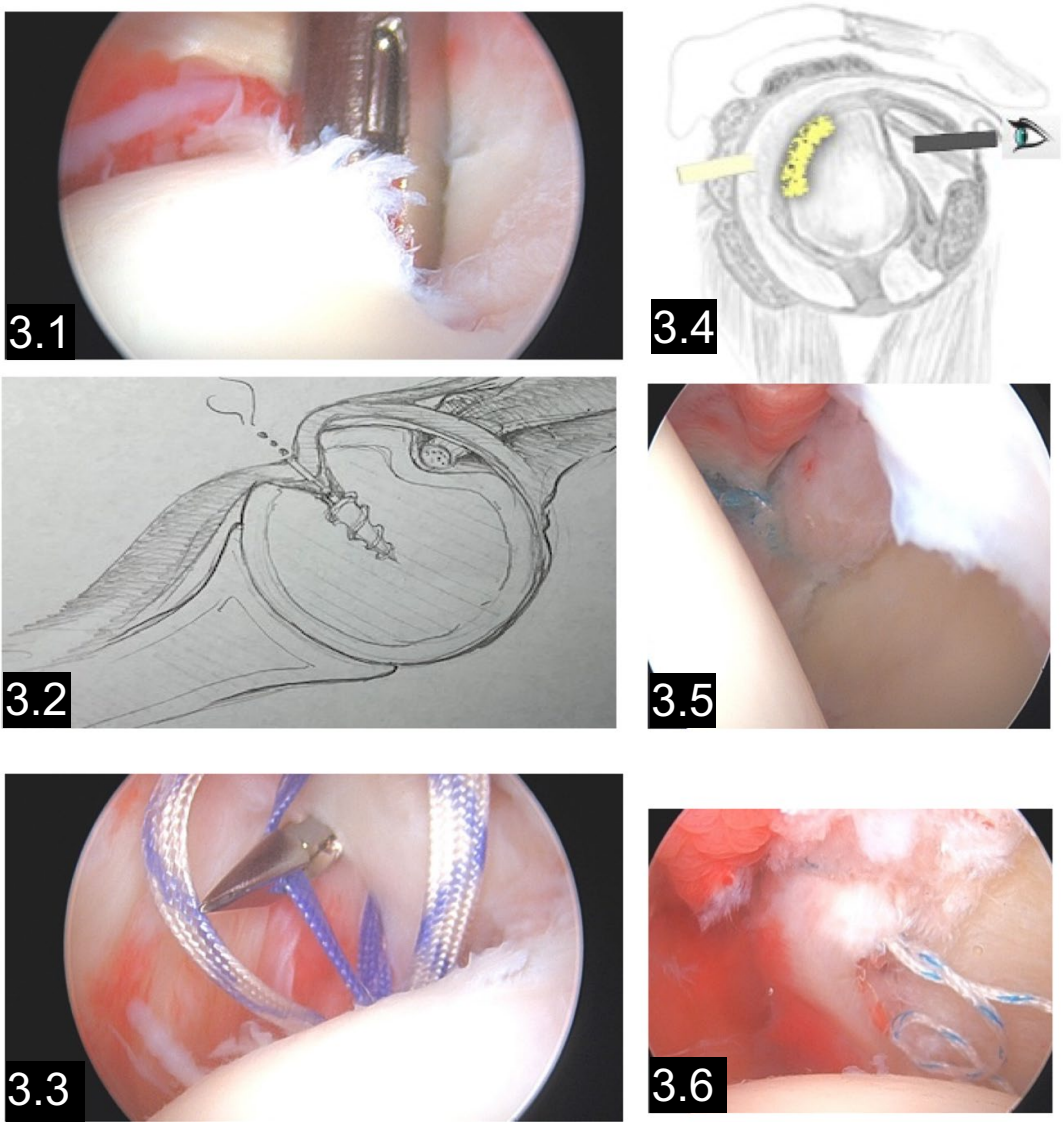
Illustrates the positioning of the suture anchor to the reversed Hill Sachs lesion on the anterior humeral head. Figure 3.2 The first schematic illustration demonstrates the Remplissage procedure by pulling the subscapularis tendon to the bony defect, therefore limiting the range of motion in internal rotation, and protecting the humeral head to subluxate in internal rotation. Figure 3.3 shows a straight bird peek stitching through the subscapularis tendon grabbing the sutures placed in the reversed hill Sachs lesion. Figure 3.4 shows the transition of the view from anterior to posterior and the second injury of a labral tear Fig. 3.5–6 illustrate in the same patient the refixation of the dorsal labral tissue to the glenoid rim. The arthroscope is inserted from the frontal portal

**Fig. 4:**
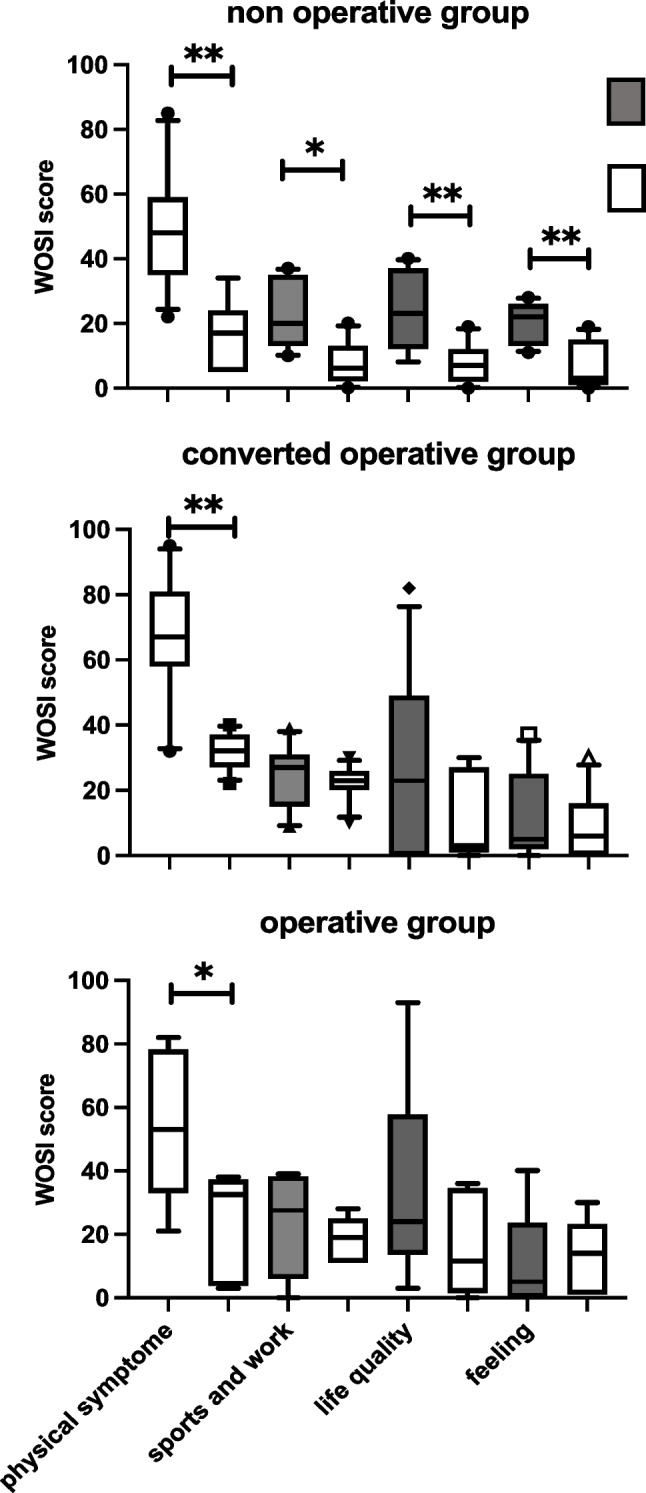
Shows our results dark grey before and white box after treatment graphically using 3 box plot diagrams, for each group one. The first plot visualizes the conservative treated patients, the second the converted, and the third the primarily operative treated group of patients. The illustrated WOSI score is divided into physical symptoms, sports and work, life quality, and feeling. One star * illustrates *α* > 0.05 and two ** 0.001. There is a significant difference in all questions in the conservative-treated group, but only in the physical symptoms questionary in the second and third graph before and after treatment.

Considering the entire cohort, the patient assessments before treatment resulted in an average WOSI score of 127 points. After therapy, this score improved to an average of 55 points, signifying an overall subjective improvement of 43%. To provide a more detailed evaluation of the WOSI score, the results of the arthroscopically and conservatively treated groups were analyzed separately. In the group primarily designated for conservative treatment from the surgeon’s perspective, the WOSI score improved from 122 to 57 points. Conversely, in the group initially designated for operative treatment, the WOSI score decreased from 133 to 53 points (Fig. 4).

## Discussion

Decision-making in the management of traumatic posterior shoulder dislocation and subsequent posterior shoulder instability (PSI) is a complex process with varying approaches. On one hand, researchers like Blacknall and Moroder have shown good results with conservative therapy [7, 19]. On the other hand, Cruz-Ferreira et al. established the superiority of arthroscopic therapy for PSI in their direct comparison of treatment options [22]. Considering this discrepancy, our retrospective study aimed to directly compare conservative and operative treatments and analyze our therapy algorithm, although our initial intention was not exclusively arthroscopic treatment.

Our case–control study demonstrates that nonoperative treatment is a recommended approach for traumatic posterior shoulder dislocation, particularly in cases without a direct surgical indication and low initial clinical complaints, as indicated by a low Western Ontario Shoulder Instability Index (WOSI) score. Overall, 25 out of the 28 (89%) patients benefited from the treatment and could be classified as having achieved therapeutic success. We considered a clinical therapeutic success to be present when an improvement of at least 10.4% in the WOSI score was recorded, consistent with the recommendation of Kirkley et al. [23]. The success rate in the group of patients treated conservatively did not significantly differ from that of the group undergoing surgical treatment (*p* = 0.980). Among the patients treated with arthroscopic procedures, the average WOSI score dropped from 133 points before treatment to 53 points after the operation, representing an 80-point improvement. Of the 17 patients treated arthroscopically, 16 achieved successful outcomes, with ten of them having previously attempted conservative therapy. Conversely, patients who received conservative treatment improved their WOSI scores from an initial average of 122 points to 57 points (Table 1).

**Table 1:** Demographic and physical examination data for all patients, including type of injury and treatment in relation to pre- and post-treatment WOSI assessment

	Patient no	Age	Sex	Cause	Damage/lesion	ABC classification	Conservative attempt	Way of treatment/arthroscopic method	WOSI at pretreatment	WOSI at posttreatment	Difference in %
Arthroscopic	1	27	M	Unknown	Rev. Bankart/SLAP	B2	X	Posterior Bankartrefixation	139	116	− 17%
	2	26	M	Trauma	Rev. Hill-Sachs/rev. Bankart	A2		Posterior Bankartrefixation	133	22	− 83%
	3	49	M	Trauma	Rev. Hill-Sachs/rev. Bankart	A2	X	Reconstruction of the glenohumeral ligaments	150	34	− 77%
	4	43	M	Trauma	Bankart/Hill Sachs/SLAP	A2	X	Posterior Bankartrefixation	103	12	− 88%
	5	40	M	Trauma	Hill-Sachs	A2	X	Reconstruction of the impression fracture	163	11	− 93%
	6	26	M	Trauma	Reverse Bankart	A2	X	Posterior capsular tightening and bone block	165	39	− 76%
	7	26	F	Trauma	Pulley/HAGL/rev. Bankart	A2	X	Posterior Bankartrefixation	201	179	− 11%
	8	40	F	Trauma	SLAP/rev. Bankart	A2	X	Remplissage + fixation of the humeroglenoid capsule ligaments	150	113	− 25%
	9	21	M	Trauma	Reverse Bankart	A2		Posterior Bankartrefixation and gathering of the IGHL	52	4	− 92%
	10	21	M	Trauma	Reverse Bankart	A2	x	Refixation of the humeroglenoid capsule ligaments	155	0	− 100%
	11	20	M	Trauma	Reverse Bankart	A2		Remplissage + posterior Bankartrefixation	43	38	− 12%
	12	20	M	Trauma	Reverse Bankart	A2		Posterior Bankartrefixation and gathering of thet rear capsule	146	28	− 81%
	13	17	F	Trauma	Reverse Bankart	B2		Posterior Bankartrefixation and gathering of thet rear capsule	178	81	− 54%
	14	28	M	Trauma	Reverse Bankart	A2	X	Reconstruction of the glenohumeral ligaments	96	15	− 84%
	15	52	M	Trauma	Reverse Bankart	A2		Posterior Bankartrefixation	124	101	− 19%
	16	29	M	Trauma	SLAP/rev. Bankart	A2	X	Posterior Bankartrefixation	135	0	− 100%
	17	41	F	Chronical	Hill-Sachs/SLAP	B1		Reconstruction of the glenohumeral ligaments	129	110	− 15%
	Average:								133	53	− 60%
Conservative	18	51	F	Trauma	Hypoplastic ant./post. labrum	A2		Physiotherapy	179	199	11%
	19	17	M	Microtrauma	Unknown	A1		Training the shoulder muscles for recentering	83	17	− 80%
	20	36	M	Trauma	Hill-Sachs/joint effusion	A2		Physiotherapy and manual therapy with NSAR	158	71	− 55%
	21	35	M	Trauma	Unknown	A2		NSAR/kryotherapy	138	5	− 96%
	22	44	F	Trauma	Tossy-damage/distortion	A2		NSAR/Tilidin/physiotherapy	187	78	− 58%
	23	36	M	Trauma	Hill-Sachs	A2		Infiltration/NSAR/physiotherapy	93	36	− 61%
	24	23	M	Trauma	Unknown	A2		NSAR/physiotherapy/infiltration	86	79	− 8%
	25	26	F	Trauma	Rev. Hill-Sachs/rev. Bankart	A2		NSAR/kryotherapy/immobilization with Gilchrist sling	91	8	− 91%
	26	24	M	Trauma	Unknown	A2		NSAR/physiotherapy	98	63	− 36%
	27	53	M	Trauma	Reverse. Hill-Sachs	A2		Cortisone shock therapy	172	53	− 69%
	28	55	M	Trauma	Capsule damage/joint effusion	A2		NSAR/kryotherapy/physiotherapy	56	20	− 64%
								Average:	122	57	− 53%

These results align with our initial hypothesis that conservative therapy, when indicated, can yield positive outcomes comparable to those achieved through operative therapy. Therefore, immediate surgical intervention cannot be justified, given the associated risks relative to conservative treatment. While the improvement in the WOSI score was greater in the operative treatment group, it is important to note that these patients typically began therapy with more severe trauma and higher initial WOSI scores. Nevertheless, only two patients in our study did not show significant improvement. One patient, numbered 24, who was treated conservatively, only experienced an 8% improvement. The exact reasons for this treatment failure remain unknown. In contrast, patient 18, initially treated conservatively, saw a marginal increase in their WOSI score from 179 to 199 points, representing an 11% difference. Patient seven, with an initial score of 201 points, received arthroscopic treatment, which resulted in a lower score of 179 points, also reflecting an 11% difference and representing an unsatisfactory outcome.

An analysis of therapy failures indicates that patient 18 refused arthroscopic surgery and opted for conservative treatment. After a telephone consultation, she reported worsening symptoms and hand numbness while cycling. In the case of patient seven, multiple arthroscopic procedures were performed, which, according to studies by Jain et al. have been associated with worse outcomes in patients with nontraumatic PSI [18, 24]. An error rate of 11% in our study is consistent with the average reported in comparable studies, including the Norwegian Register for Shoulder Instability Surgery and the systematic review by Leivadiotou et al. which documented a mean recurrence rate of 5.51% among 387 patients with PSI [2, 17]. An analysis of WOSI scores was conducted using paired *t* tests on different patient subgroups to compare the scores of arthroscopically treated patients with those who received conservative treatment. This analysis revealed a significant difference with *p* < 0.05.

In our study, we confirm the currently recommended arthroscopic treatment for patients with bone damage, and our arthroscopic cohort demonstrates excellent results. To our knowledge, only one study has directly compared arthroscopic and conservative treatments. Cruz-Ferreira et al. concluded that operative treatment yielded better outcomes than nonoperative therapy, though this conclusion was primarily based on slightly improved scoring systems [22]. Our study design was not explicitly aimed at comparing therapy alternatives directly, as it is a two-arm case–control study for PSI, with treatment changes occurring in ten out of 28 patients within the first three months. Our primary focus was the initial decision-making process, rather than the intention to directly compare treatment options.

In Cruz-Ferreira’s study, the comparison of therapy options showed that in the nonoperative and operative groups, the Constant score was 78 versus 87, the Rowe score was 64 versus 88, and the Walch-Duplay score was 69 versus 82, which closely mirrors our results [22]. However, the risks associated with operative intervention were not fully considered by Cruz-Ferreira et al. when assessing treatment options. Our conclusion supports the idea that conservative therapy can offer similar success with fewer risks in patients without absolute operative indications and low initial subjective complaints, as reflected in the WOSI score.

Minor structural injuries following PSI, such as capsule ruptures or minor SLAP lesions, can often be effectively treated with physiotherapy, specifically focused on muscle strength regain. According to a biomechanical theoretical study by Hölscher et al. the shoulder joint is primarily guided by muscles, and the humeral head is centered by the shoulder reaction force produced predominantly by the rotator cuff muscles [8]. Training these shoulder muscles to redirect the force toward the centre can support the healing process in cases of posterior shoulder complex trauma involving labral and capsule injuries [8]. The results of Moroder et al. who achieved positive outcomes using EMS training to strengthen shoulder muscles in patients with functional posterior shoulder instability, reinforce the efficacy of this approach [10]. In line with our findings, the study by Blacknall et al. demonstrates that specialized physiotherapy rehabilitation is a valuable treatment option for atraumatic posterior shoulder instability, resulting in significant clinically important improvements in patient-reported outcomes [7].

In our study, the Western Ontario Shoulder Instability Index (WOSI) was employed as a reliable instrument to measure treatment success, which is independent of the examining physician. Furthermore, the “Core Outcome Measures in Effectiveness Trials” (COMET) suggests that future shoulder studies should encompass pain, physical function, the overall assessment of therapy success, and health-related quality of life as their main categories [25]. The WOSI covers three of these four main criteria: pain, physical functionality, and health-related quality of life. It also provides the most accurate recording of subjective symptoms and is known for its reliability, validity, and accuracy [25, 26]. The greatest advantage of the WOSI is its ability to measure shoulder instability in relation to an individual’s quality of life, which is why it was selected for this study.

Many studies have suggested a correlation between an increased glenoid retroversion and the risk of developing dynamic PSI. For instance, Galvin et al. found that patients with posterior glenohumeral instability (PGHI) exhibited an average glenoid retroversion of − 8.16° during MRI analysis, whereas the control group had a retroversion of − 2.9° [27]. Owens et al. took this concept further by postulating that for every degree of increased retroversion, the risk of PSI increased by 17% [28]. In contrast, Yoo et al. found no significant correlation between PSI and glenoid retroversion [29]. In our study cohort, we measured an average glenoid retroversion of − 4.3° using the Friedman method and an average retroversion of − 17.53° using the “glenoid vault” method. A deviation of 6.3° in the Friedman method falls within acceptable measurement tolerances, as confirmed by Friedman et al. who reported a range of − 12 to 14° in their control cohort [19]. This suggests that the examined patient cohort did not exhibit osteoarthritic changes in the glenoid, which, in other studies, could be linked to increased retroversion [27, 28, 30]. We concluded that the PSI in our cohort primarily originated from traumatic events. Given the relatively low prevalence of PSI, accounting for approximately 10% of all shoulder instabilities, the sample size is insufficient to achieve significant statistical significance. Additionally, the study design only allowed for treatment observation, and group assignments were determined retrospectively.

Ethically, it is debatable whether patients requiring immediate surgery and those who may not need surgery can be included in a comparative study. Our observational study leaves the question open regarding whether patients with a WOSI score below 125 might experience even greater improvement with surgery.

The results of this study are in line with the existing scientific knowledge, suggesting that the best initial approach is to initiate a three-month trial of nonoperative therapy when there are no absolute surgical indications [3, 31, 32]. If nonoperative therapy proves unsuccessful, then secondary surgery, particularly in cases with clear surgical indications, is recommended. For post-traumatic PSI without significant hard tissue defects, which still retains the ability to actively stabilize the shoulder, nonoperative therapy should be the primary choice, unless the WOSI score is higher than 130. In the case of shoulder injuries in performance athletes, primary reconstruction via arthroscopy is preferable [14].

## Conclusion

Patients diagnosed with posterior shoulder instability (PSI) presenting with a Western Ontario Shoulder Instability Index (WOSI) score exceeding 50 warrant a comprehensive examination to assess the extent of physical damage. In cases where substantial damage to bone, cartilage, and the rotator cuff, along with subluxation, is evident, surgical intervention should be the primary course of action. Conversely, in patients with a glenohumeral joint that centers in rest and a WOSI score below 125, a conservative therapy trial of three months should be initiated. The emphasis during this phase should be on recentering the humeral head within the joint socket through muscular strengthening. Notably, both conservative and operative treatments have demonstrated the potential for substantial improvements when the treatment indication is appropriate.

## Data Availability

The data that support the findings of this study are available from the corresponding author, Carsten Englert, MD, upon reasonable request.
